# CancerGram: An Effective Classifier for Differentiating Anticancer from Antimicrobial Peptides

**DOI:** 10.3390/pharmaceutics12111045

**Published:** 2020-10-31

**Authors:** Michał Burdukiewicz, Katarzyna Sidorczuk, Dominik Rafacz, Filip Pietluch, Mateusz Bąkała, Jadwiga Słowik, Przemysław Gagat

**Affiliations:** 1 Faculty of Natural Sciences, Brandenburg University of Technology Cottbus-Senftenberg, 01968 Senftenberg, Germany; michalburdukiewicz@gmail.com; 2 Why R? Foundation, 03-214 Warsaw, Poland; dominikrafacz@gmail.com; 3 Department of Bioinformatics and Genomics, Faculty of Biotechnology, University of Wrocław, 50-383 Wrocław, Poland; katarzyna.sidorczuk2@uwr.edu.pl (K.S.); filip.pietluch2@uwr.edu.pl (F.P.); 4 Faculty of Mathematics and Information Science, Warsaw University of Technology, 00-662 Warsaw, Poland; matibakala@gmail.com (M.B.); jadwigaslowik5@gmail.com (J.S.)

**Keywords:** anticancer peptide (ACP), antimicrobial peptide (AMP), anticancer peptides, antimicrobial peptides, host defense peptides, prediction, random forest

## Abstract

Antimicrobial peptides (AMPs) constitute a diverse group of bioactive molecules that provide multicellular organisms with protection against microorganisms, and microorganisms with weaponry for competition. Some AMPs can target cancer cells; thus, they are called anticancer peptides (ACPs). Due to their small size, positive charge, hydrophobicity and amphipathicity, AMPs and ACPs interact with negatively charged components of biological membranes. AMPs preferentially permeabilize microbial membranes, but ACPs additionally target mitochondrial and plasma membranes of cancer cells. The preference towards mitochondrial membranes is explained by their membrane potential, membrane composition resulting from α-proteobacterial origin and the fact that mitochondrial targeting signals could have evolved from AMPs. Taking into account the therapeutic potential of ACPs and millions of deaths due to cancer annually, it is of vital importance to find new cationic peptides that selectively destroy cancer cells. Therefore, to reduce the costs of experimental research, we have created a robust computational tool, CancerGram, that uses *n*-grams and random forests for predicting ACPs. Compared to other ACP classifiers, CancerGram is the first three-class model that effectively classifies peptides into: ACPs, AMPs and non-ACPs/non-AMPs, with AU1U amounting to 0.89 and a Kappa statistic of 0.65. CancerGram is available as a web server and R package on GitHub.

## 1. Introduction

There are many health care issues that challenge the welfare of humankind; among them, cancer and antimicrobial resistance are of ever-growing concern. According to the World Health Organization, cancer is a leading cause of death globally, responsible for about 9.6 million deaths in 2018 [1], and antimicrobial resistance threatens our ability to treat an increasing number of infectious diseases, with a death toll of tens of thousands of people in Europe and the United States [2,3]. Interestingly, both these challenges could be approached with cationic peptides, antimicrobial peptides (AMPs) and anticancer peptides (ACPs), respectively.

AMPs, also known as host defense peptides, constitute a diverse group of bioactive molecules that provide multicellular organisms with protection against bacteria, fungi, protozoans and viruses [4,5], and microorganisms with weaponry for competition [6,7]. Some AMPs can target cancer cells; this particular group of AMPs is called anticancer peptides (ACPs). AMPs, including ACPs, are short peptides, generally with fewer than 50 amino acids, that are rich in positive and hydrophobic residues, and, consequently, have amphiphilic properties [4,8]. Due to these characteristics, they preferentially interact with negatively charged components of biological membranes, which are typical of the bacterial cell wall and the plasma membrane of cancer but not healthy cells. As a result, AMPs and ACPs lead to membrane micellization and/or permeabilization by forming pores [9,10,11,12]. By definition, AMPs target microbial membranes, especially bacterial envelopes, but ACPs, apart from their antimicrobial activity, also exhibit anticancer properties due to slightly different amino acid composition (for details, see [13] and Section 3.1).

One of the promising targets of anticancer therapies are mitochondria, cytoplasmic organelles derived from an ancestor of α-proteobacteria [14,15,16]. Mitochondria not only provide the energy and building blocks for new cells, but they are also the regulatory centers of redox homeostasis and apoptosis [17]. Interestingly, ACPs can bind to and affect the integrity of the plasma membrane of cancer cells; however, they preferentially disrupt mitochondrial membranes—specifically, they do so at concentrations hundreds of times lower than the concentrations for plasma membrane disruption [18]. The preference is due to the difference in membrane potential that is generated during oxidative phosphorylation at the inner mitochondrial membrane by proton pumps [19]. The membrane potential drives cations and cationic peptides into mitochondria, but because it is steadily increased in cancer cells, it provides even greater killing capacity in the cancerous environment [19,20,21]. Some preference for targeting mitochondria is also attributed to the fact that mitochondrial membranes still resemble, in terms of composition, the envelope of Gram-negative bacteria, and, therefore, attract AMP-like molecules [22,23]. Moreover, proteins imported into mitochondria carry an N-terminal targeting signal known as the mitochondrial transit peptide, which actually could have evolved from AMPs [24,25]. Since mitochondrial transit peptides show considerable similarity to their presumed progenitors, AMPs might also use the traditional Tom/Tim-dependent pathway to enter mitochondria [26].

Taking into account the therapeutic potential of ACPs, i.e., high target specificity, good efficacy, low toxicity, easy chemical modification and synthesis, it is of vital importance to find new cationic peptides that could target cancer cells [12,27]. Unfortunately, the experimental procedures to identify novel ACPs are time-consuming and expensive. Consequently, there is a demand for efficient and accessible bioinformatics tools that could help indicate potential ACP candidates with high accuracy for further research.

A number of computational approaches have been adopted for ACP prediction; however, there are serious concerns about the quality and quantity of sequences that were used for their development [13]. As a result, these algorithms have problems to discriminate between peptides with similar composition but different activity, i.e., between AMPs and ACPs. Some do not also provide web servers, and, therefore, have limited utility for biologists not well acquainted with bioinformatics ([13] and citations therein).

Our goal was to create a robust three-class model, CancerGram, for differentiating ACPs from AMPs and sequences that are neither ACPs nor AMPs. CancerGram uses *n*-grams (continuous or discontinuous sequences of *n* elements) and random forests (a machine learning method) for the classification algorithm. *N*-grams represent short motifs that are relevant to anticancer, antimicrobial and non-anticancer/non-antimicrobial properties of peptides, and they allow us, in an easily interpretable way, to discriminate between the three classes of molecules. This methodology has already been used with success in our previous projects to develop software for predicting AMPs [28], amyloid proteins [29] and signal peptides [30], and to assess the optimal growth conditions for methanogens [31]. CancerGram addresses the above-mentioned shortcomings of other ACP classifiers using verified data sets from AntiCP 2.0, which is the top-ranking ACP predictor [13]. However, compared to AntiCP 2.0, the decision making process of CancerGram is performed at the same time between three classes of sequences, i.e., ACPs, AMPs and non-ACPs/non-AMPs; therefore, it is convenient from the point of view of the user.

## 2. Materials and Methods

### 2.1. Data Sets

The data sets used to develop CancerGram were acquired from Agrawal et al. [13]. The training and validation data sets contained, respectively, 689 and 172 experimentally verified ACPs, 689 and 172 AMPs without anticancer activity and 776 and 194 non-ACP/non-AMP sequences (the negative data set). After the removal of peptides shorter than 5 amino acids, the data sets were used for CancerGram training and validation of its performance. The final numbers of sequences in each class are presented in Table 1. Since we could not repeat the benchmark analyses for AntiCP 2.0 [13], to compare its performance with CancerGram, we downloaded 2952 experimentally verified ACPs from CancerPPD [32], APD3 [33] and DRAMP [34] database and 4118 AMPs from dbAMP database [35]. We removed the most similar sequences using CD-HIT [36], assuming 0.95 and 0.60 identity threshold for ACPs and AMPs, respectively. Next, we removed sequences that were already contained in the training and validation data sets of CancerGram and AntiCP 2.0 [13]. As a result, we obtained an unbiased data set, termed independent, containing 57 ACPs and 769 AMPs (Table 1).

### 2.2. Cross-Validation

**Figure 1:**
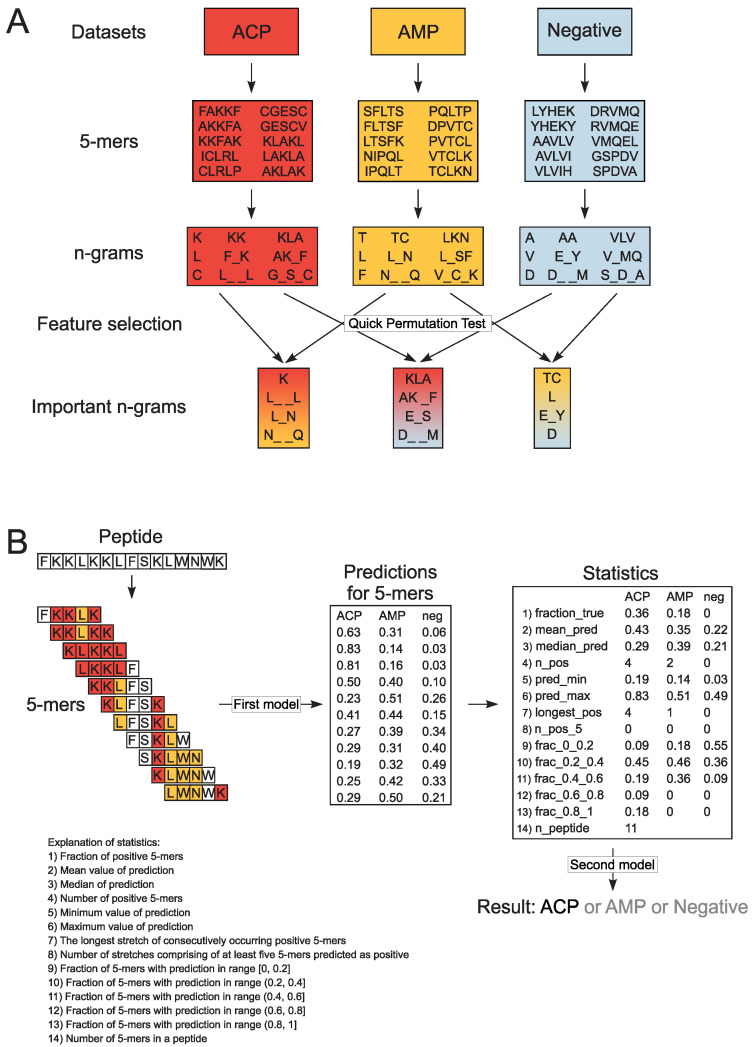
Schematic representation of *n*-gram extraction (**A**) and decision-making procedure in CancerGram (**B**). The training data sets include ACP (shaded in red), AMP (shaded in yellow) and non-ACP/non-AMP sequences (the negative data set, shaded in blue). Each peptide from the training data sets was divided into subsequences of 5 amino acids (5-mers). For each 5-mer, we extracted continuous and discontinuous *n*-grams of size ranging from 1 to 3, and exemplary *n*-grams are presented in boxes shaded in colors respective to the data sets. The informative *n*-grams for CancerGram training were selected by Quick Permutation Test for all combinations of the data sets, and they are shaded in: (i) red-yellow for the ACP/AMP data set, (ii) red-blue for the ACP/Negative data set, and (iii) yellow-blue for the AMP/Negative data set (**A**). To make a prediction, CancerGram first divides a peptide into 5-mers and then, for each 5-mer, makes a prediction if it is an ACP, AMP or non-ACP/non-AMP (the first model). To scale the prediction from 5-mers to the level of a peptide, numerous statistics are calculated, and on their basis, CancerGram makes the final prediction (the second model) (**B**).

We divided the ACP, AMP and non-ACP/non-AMP training data sets into five groups (folds), ensuring approximately the same sequence length distribution in each group for each data set. Next, we performed the fivefold cross-validation on both the mer and peptide layers of the model (for details, see Section 2.4 and Figure 1). The results of the cross-validation are presented in Table 2 and Figure 4.

### 2.3. Extraction of Encoded N-Grams

In order to create the three-class model, we divided each sequence from the training data sets into overlapping subsequences of 5 amino acids (5-mers); the length of 5 amino acids represents the shortest ACPs in our data sets. Consequently, we obtained 11,496 ACP, 15,826 AMP and 18,587 non-ACP/non-AMP 5-mers. From each 5-mer, we extracted *n*-grams, i.e., sequences of *n* elements. We analyzed continuous and discontinuous *n*-grams of size ranging from 1 to 3. In the case of discontinuous *n*-grams, bigrams (*n*-grams of size 2) could contain a gap of length from 1 to 3 (e.g., L_N, C_ _G, K_ _ _K), whereas in trigrams (*n*-grams of size 3), there is only a single gap between the first and the second and/or the second and the third amino acid (e.g., K_L_L, AK_F, L_SA). The gap corresponds to the presence of any amino acid. The *n*-gram presence was then counted and binarized for each 5-mer. The binarization of *n*-grams means that if an *n*-gram is present (at least once) in the 5-mer, it obtains the value of 1, and 0 if it is absent (Figure 1A).

### 2.4. Model Training with Random Forests

To select the informative *n*-grams, we performed Quick Permutation Test (QuiPT) [37] on each combination of classes (ACP/AMP data set, ACP/Negative data set and AMP/Negative data set) with *p*-value threshold 0.0001. We obtained 1883 informative *n*-grams and used them for CancerGram training. We trained the first random forest model on binarized occurrences of informative *n*-grams in 5-mers using the ranger R package [38]. The number of trees was set to 2000 and mtry parameter, i.e., the number of variables randomly sampled as candidates at each split, to the default value.

In order to scale the information found in 5-mers to the level of a peptide, we calculated numerous statistics for each peptide and for each class (Figure 1B) according to the methodology used in our previous projects [28]. These statistics were subsequently used to train the second random forest model predicting the class of a given peptide (ACP, AMP or non-ACP/non-AMP). In this case, both the mtry parameter and number of trees (500) were set to the default values. Consequently, the model is composed of stacked random forests [39], where the first one evaluates the probability of each 5-mer derived from a peptide as ACP, AMP or non-ACP/non-AMP, and the second considers statistical results for all mers from the given peptide and decides whether the whole peptide is ACP, AMP or non-ACP/non-AMP (Figure 1B).

## 3. Results and Discussion

### 3.1. Composition and Properties of ACPs and AMPs

**Figure 2:**
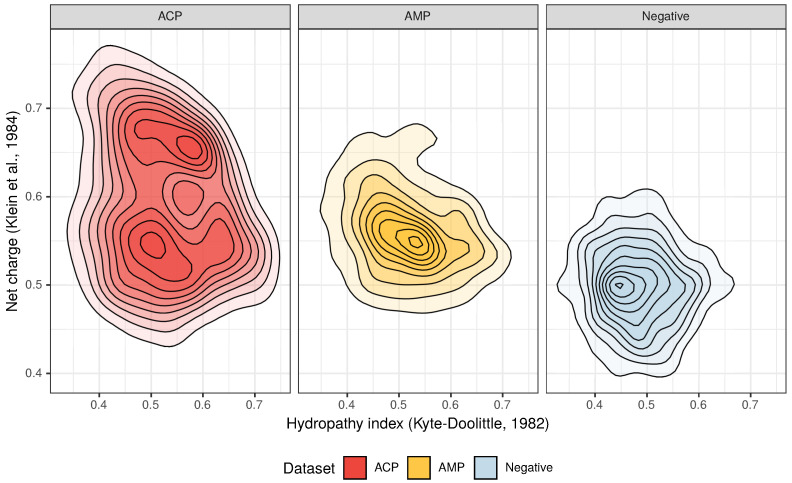
Distribution of the hydropathy index and net charge for anticancer peptides (ACPs), antimicrobial peptides (AMPs) and non-ACP/non-AMP sequences (Negative).

**Figure 3:**
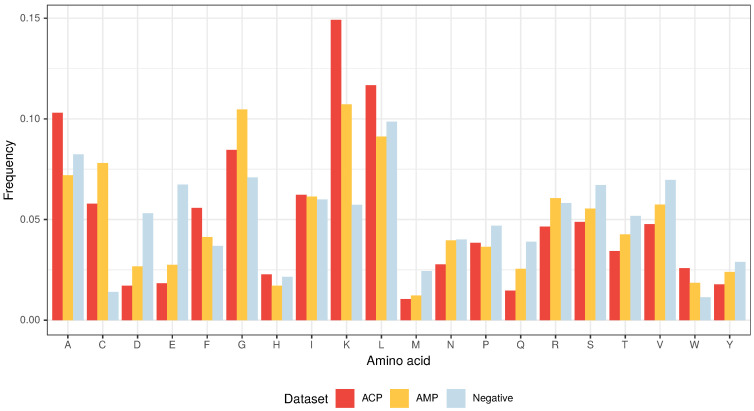
Amino acid composition of ACPs, AMPs and non-ACP/non-AMP sequences (Negative).

The amino acid composition that characterizes both ACPs and AMPs (Figure 2 and Figure 3) defines their properties, such as positive charge, hydrophobicity and amphipathicity, and they, in turn, determine their propensity for damaging bacterial and cancer cell membranes [40]. First, the positively charged molecules are driven electrostatically to the negatively charged membranes, and then their hydrophobicity and amphipathicity allows them to penetrate into the membrane and destabilize it in a detergent-like manner (carpet model) and/or by forming pores (barrel-stave or toroidal model) [9,10,11,12].

From the three above properties, only the positive charge differentiates the ACP group from AMPs because the upper limit of the positive charge is elevated for ACPs (Figure 2). This is the result of a high frequency of lysine (K), which is a predominant amino acid component of ACPs [13]. Interestingly, arginine (R), which is another basic amino acid, is slightly depleted in ACPs in comparison with AMPs and peptides from the negative data set (Figure 3). The decrease in arginine residues may, however, be beneficial for ACPs as its side chain, compared to lysine’s, exhibits higher affinity for zwitterionic (neutral) membranes of healthy cells, and, therefore, is much more toxic [27].

Apart from its positive charge, lysine is also hydrophobic in nature and, as stated above, the hydrophobicity is another important property of both ACPs and AMPs. Peptides with higher hydrophobicity could be able to penetrate deeper into the hydrophobic core of the cell membrane, and, consequently, exhibit stronger propensity to permeabilize it [41]. ACPs are much richer in lysine (K), leucine (L), alanine (A) and phenylalanine (F) compared to AMPs and the peptides from the negative data set (Figure 3) [13]. In addition to its rather weak hydrophobic properties, alanine is also a good helix-forming residue; ACPs are known to form α-helical structures [40]. The last hydrophobic amino acid that deserves attention, tryptophan (W), is generally rare in proteins, but there seems to be more of it in ACPs compared to the other analyzed data sets though it is not statistically significant (Supplementary Tables S1–S3). Tryptophan serves an important role by helping peptides enter cancer cells via the endocytic pathway, thereby traversing the plasma membrane [42,43].

The other two amino acids that are abundant in ACPs, but not as much as in AMPs, are glycine (G) and cysteine (C) (Figure 3). The former is known to provide peptides with conformational flexibility and the latter to stabilize and maintain their proper motif and domain structure [43].

Although ACPs and AMPs are generally considered to be similar in terms of properties and the mode of action, the differences in their amino acid composition are significant enough (Supplementary Tables S1–S3) to find informative motifs that characterize them and non-ACPs/non-AMPs, thereby training an effective model for predicting ACPs.

### 3.2. CancerGram Performance

**Figure 4:**
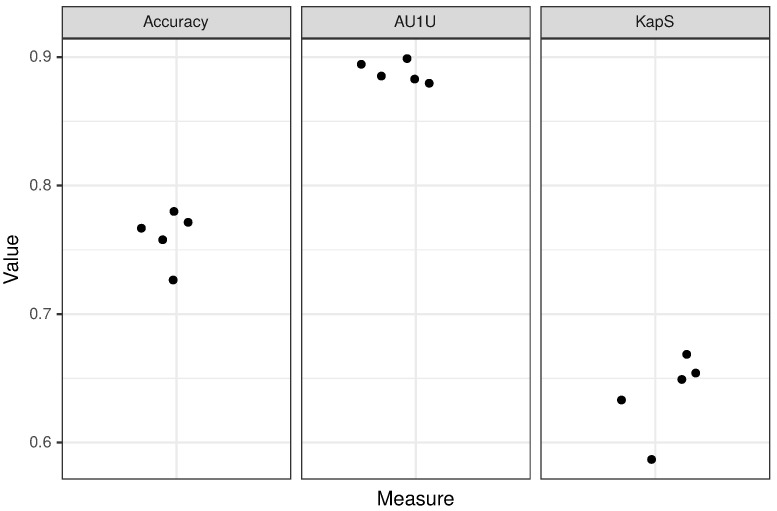
Results of fivefold cross-validation for the peptide layer of the model. Each dot corresponds to a single fold.

In order to evaluate the performance of CancerGram, we have chosen three measures: (i) accuracy, (ii) mean AUC (area under the ROC curve) for binary comparisons of each class against each other (AU1U) and (iii) Kappa statistic (KapS) [44]. Accuracy is the simplest and the most common measure to evaluate the performance of a classifier. In the case of CancerGram, it simply provides the fraction of well-predicted ACPs, AMPs and non-ACPs/non-AMPs. A better measure is AU1U, the approximation of AUC for multi-class models. It informs the user of how much the model is able to distinguish between the three classes of peptides, i.e., ACPs, AMPs and non-ACPs/non-AMPs. A more general interpretation is that AU1U represents the probability that, e.g., a randomly selected ACP will be ranked higher in the ACP class than a random AMP or non-ACP/non-AMP. The values of both accuracy and AU1U range from 0 to 1, where 0.5 means a useless, i.e., a random classifier [45]. The last measure used to evaluate CancerGram is KapS, and it contains the information about how much better the model performs compared to the classifier that simply guesses at random according to the number of elements in each class. KapS evaluates the degree of agreement between CancerGram predictions and the true labels [46]. It takes values in [−1, 1], where 0 means a random classifier and values above 0.80 indicate an excellent one [47]. All measures were calculated using the measures R package [48]. The results of CancerGram validation are presented in Table 3 and the results of the fivefold cross-validation are presented in Table 2 and Figure 4.

CancerGram is a robust model with AU1U amounting to 0.89. The value of KapS 0.65 (0.64 for fivefold cross-validation) informs us that CancerGram is a good model [47]. The least informative measure for the three-class model is the accuracy because, among other things, it does not take into account the distribution of the misclassification among classes, and it is equal to 0.77 (0.76 for fivefold cross-validation). From the point of view of the researcher interested in screening for ACPs, the most important issue is the restrictiveness of the model in terms of false ACP predictions. Accordingly, CancerGram falsely identifies only 1.5% of the non-ACPs/non-AMPs as ACPs (3 out of 194 from the validation data set) and less than 16% AMPs (27 out of 170 from the validation data set).

CancerGram is not only an effective model for ACPs prediction but also the only three-class model available at present. The other ACP classifiers represent binary models, and they have problems with distinguishing between sequences with similar amino acid composition but different activity, i.e., ACPs and AMPs [13]. AntiCP 2.0 has overcome the problem; however, the greatest disadvantage of AntiCP 2.0 is that the biologist may become confused about which model they should use from the ones available on the AntiCP 2.0 web server. The first one is a binary model that differentiates between ACPs and AMPs, and the second between ACPs and non-ACPs [13].

In order to compare the CancerGram and AntiCP 2.0 [13] performance, we decided to test their predictive power towards classification of ACPs and AMPs, which is most challenging for ACP predictors [13]. Interestingly, we could not use the validation data set bacause the final version of AntiCP 2.0 [13] was possibly trained not only on the training but also the validation data set; we were not able to repeat their benchmark analyses. Therefore, we constructed an independent data set containing 57 ACP and 769 AMP sequences. Since CancerGram is a three-class model, we had to binarize its prediction, i.e., the prediction results for AMPs and non-ACPs/non-AMPs were summed and represent the AMP class. CancerGram outperformed AntiCP 2.0 [13] in terms of AUC, accuracy, specificity and the Matthews correlation coefficient (MCC) (Table 4). Sensitivity and specificity indicate the proportion of ACPs and AMPs that were correctly identified as ACPs and AMPs, respectively. Precision reflects the proportion of predicted ACPs that are truly ACPs, and MCC represents a reliable metric for binary classifiers, i.e., a balanced measure of correlation coefficient between predictions and true labels. We also compared the performance of CancerGram with mACPpred [49] because it has recently been published but not included in Agrawal et al. [13] as the benchmark on the validation data set. The mACPpred model, similarily to AntiCP 2.0 [13], is also not as robust as CancerGram and, moreover, compared to AntiCP 2.0 [13] and CancerGram, it tends to predict AMPs as ACPs, i.e., it generates numerous false positive results (low specificity) (Table 5).

### 3.3. Prediction of Mitochondria-Targeted ACPs with CancerGram

We also wanted to check the predictive power of CancerGram toward ACPs that have been experimentally verified to target mitochondria of cancer cells. By searching the literature, we did find 12 ACPs that were not included in our training data sets (Table 6). The results of the analysis are presented in Table 7. As expected, CancerGram correctly identified most of them, i.e., eight sequences, although it identified GW-H1, lactoferricin B and pleuricidin NRC-03 as AMPs, and A${}_{9}$K as a non-ACP/non-AMP.

## 4. Conclusions

Based on data sets from Agrawal et al. [13], we have compared ACPs, AMPs and non-ACP/non-AMP sequences in terms of their amino acid composition. In the case of ACPs, the upper limit of the positive charge was elevated, mostly due to the high content of lysine, which is not only basic but also hydrophobic. The other residues that are overrepresented in ACPs, compared to AMPs and non-ACPs/non-AMPs, are all hydrophobic and include leucine, alanine, phenylalanine and tryptophan [13]. The positive charge, hydrophobicity and amphipathicity are responsible for AMP and ACP selectivity towards microbial membranes and, in the case of ACPs, also for targeting the cancer plasma and mitochondrial membranes. The latter are derived from α-proteobacteria and, due to their bacterial inheritance [22,23] and the potential generated during oxidative phosphorylation [18,19,20], should be preferred over the plasma membrane.

ACPs and AMPs are generally considered to be similar in terms of properties and the mode of action; however, we did find informative *n*-grams (amino acid motifs) that well differentiate them from each other and non-ACPs/non-AMPs, thereby allowing us to train an effective random forest model for ACP prediction. CancerGram is the only three-class model available at present and, moreover, it is better at discriminating between anticancer and antimicrobial peptides than other top-ranking predictors, including AntiCP 2.0 [13] and mACPpred [49]. The benchmark results also indicate that our methodology has an advantage over the methodology of Agrawal et al. [13] because, despite training our model on the same data sets, CancerGram outperformed AntiCP 2.0 on the independent data set. CancerGram is easy to use and does not require any other action other than pasting a sequence or sequences into the query box of the web server (see Appendix A). CancerGram does not predict sequences shorter than 5 amino acids, and the user should remember that it was trained on sequences up to 50 amino acids in length, i.e., it was not designed for predicting anticancer proteins.

Since new anticancer agents are desperately needed, CancerGram can be used for ACP screening to identify the best candidates for further experimental procedures. Short cationic peptides represent good antitumor agents because they are small, relatively cheap to produce and easy to modify in order to further increase their anticancer properties and stability or to lower their toxicity to healthy cells [12,27].

## Figures and Tables

**Table 1:** Data set sizes used for training and validation of CancerGram.

Data Set	ACP	AMP	Negative
Training	686	689	776
Validation	171	170	194
Independent	57	769	0

**Table 2:** Results of fivefold cross-validation.

Measure	Mer Layer	Peptide Layer
Accuracy	0.64 (+/−0.01)	0.76 (+/−0.021)
AU1U	0.79 (+/−0.006)	0.89 (+/−0.008)
KapS	0.44 (+/−0.015)	0.64 (+/−0.032)

**Table 3:** Results of predictions on the validation data sets.

Measure	Value
Accuracy	0.77
AU1U	0.89
KapS	0.65

**Table 4:** Comparison of CancerGram and AntiCP 2.0 [13] performance on the independent data set. AntiCP 2.0 predictions were obtained using model 1 of the standalone version with default values of threshold (0.5) and window length (10). CancerGram predictions were binarized. The low values of the Matthews correlation coefficient (MCC), precision and sensitivity are due to the large number of AMPs (769) and low number of ACPs (57) in the independent data set.

Software	MCC	Precision	Sensitivity	Specificity	Accuracy	AUC
CancerGram	0.15	0.17	0.30	0.89	0.85	0.60
AntiCP 2.0	0.07	0.10	0.32	0.79	0.76	0.53

**Table 5:** Comparison of CancerGram and mACPpred [49] performance on the validation data set, from which sequences used for mACPpred training were removed. The final data set contained 128 ACPs and 170 AMPs. CancerGram predictions were binarized.

Software	MCC	Precision	Sensitivity	Specificity	Accuracy	AUC
CancerGram	0.57	0.78	0.71	0.85	0.79	0.83
mACPpred	0.21	0.48	0.90	0.27	0.54	0.68

**Table 6:** Experimentally verified ACPs targeting mitochondria of cancer cells.

Peptide	Sequence	Reference
A${}_{9}$K	AAAAAAAAAK	[50]
hCAP-18	FRKSKEKIGKEFKRIVQRIKDFLRNLVPRTES	[51,52]
HPRP-A1-TAT	FKKLKKLFSKLWNWKRKKRRQRRR	[53]
KLA	KLAKLAKKLAKLAK	[54,55,56]
Lactoferricin B	FKCRRWQWRMKKLGAPSITCVRRAF	[57,58]
Magainin 1	GIGKFLHSAGKFGKAFVGEIMKS	[59]
Mastoparan-C	LNLKALLAVAKKIL	[60,61]
NGR Peptide 1	CNGRCGGKLAKLAKKLAKLAK	[56]
GW-H1	GYNYAKKLANLAKKFANALW	[62]
Pleurocidin NRC-03	GRRKRKWLRRIGKGVKIIGGAALDHL	[63]
R7-kla	RRRRRRRKLAKLAKKLAKLAK	[64]
RGD-4C-GG-(KLAKLAK)${}_{2}$	ACDCRGDCFCGGKLAKLAKKLAKLAK	[56]

**Table 7:** Prediction results for experimentally verified ACPs targeting mitochondria of cancer cells.

Peptide	ACP	AMP	Negative	Decision
A${}_{9}$K	0.10	0.32	0.58	Negative
GW-H1	0.31	0.64	0.06	AMP
hCAP-18	0.96	0.04	0.00	ACP
HPRP-A1-TAT	0.66	0.33	0.01	ACP
KLA	1.00	0.00	0.00	ACP
Lactoferricin B	0.10	0.90	0.00	AMP
Magainin 1	0.63	0.32	0.05	ACP
Mastoparan-C	0.96	0.04	0.00	ACP
NGR Peptide 1	0.65	0.35	0.00	ACP
Pleurocidin 03	0.00	1.00	0.00	AMP
R7-kla	0.96	0.04	0.00	ACP
RGD-4C-GG-(KLAKLAK)${}_{2}$	0.98	0.02	0.00	ACP

## Supplementary Materials

The following are available at https://www.mdpi.com/1999-4923/12/11/1045/s1, Table S1: Average amino acid percentages for ACPs and AMPs. The differences in amino acid composition between ACPs and AMPs were statistically evaluated using the Mann–Whitney U test with Benjamini–Hochberg correction. Table S2: Average amino acid percentages for ACPs and the negative data set. The differences in amino acid composition between ACPs and the negative data set were statistically evaluated using the Mann–Whitney U test with Benjamini–Hochberg correction. Table S3: Average amino acid percentages for AMPs and the negative data set. The differences in amino acid composition between AMPs and the negative data set were statistically evaluated using the Mann–Whitney U test with Benjamini–Hochberg correction.

## Abbreviations

The following abbreviations are used in this manuscript:

AUC	Area under the ROC curve
AU1U	AUC for binary comparisons of each class against each other
ACP	Anticancer peptides
AMP	Antimicrobial peptides
KapS	Kappa statistic
MCC	Matthews correlation coefficient
A	Alanine
R	Arginine
N	Asparagine
D	Aspartic acid
C	Cysteine
E	Glutamic acid
Q	Glutamine
G	Glycine
H	Histidine
I	Isoleucine
L	Leucine
K	Lysine
M	Methionine
F	Phenylalanine
P	Proline
S	Serine
T	Threonine
W	Tryptophan
Y	Tyrosine
V	Valine

## Appendix A. Availability and Implementation

The code necessary to reproduce the analysis presented in this paper is available in the repository: https://github.com/BioGenies/CancerGram-analysis.

The CancerGram prediction web server is available at: http://biongram.biotech.uni.wroc.pl/CancerGram/.
